# Does i-T744C P2Y12 Polymorphism Modulate Clopidogrel Response among Moroccan Acute Coronary Syndromes Patients?

**DOI:** 10.1155/2017/9532471

**Published:** 2017-02-05

**Authors:** Hind Hassani Idrissi, Wiam Hmimech, Nada El Khorb, Hafid Akoudad, Rachida Habbal, Sellama Nadifi

**Affiliations:** ^1^Laboratory of Genetics and Molecular Pathology, Medical School, University Hassan II, Casablanca, Morocco; ^2^Department of Cardiology, University Hospital Center Hassan II, Fes, Morocco; ^3^Department of Cardiology, University Hospital Center Ibn Rochd, Casablanca, Morocco

## Abstract

*Background.* An interindividual variability in response to Clopidogrel has been widely described in patients with acute coronary syndromes (ACS). The contribution of genetics on modulating this response was widely discussed. The objective of our study was to investigate the potential effect of i-T744C P2Y12 polymorphism on Clopidogrel response in a sample of Moroccan ACS patients. We tried also to determine the frequency of this polymorphism among Moroccan ACS compared to healthy subjects.* Methods and Results.* 77 ACS patients versus 101 healthy controls were recruited. DNA samples were genotyped by PCR-RFLP method. The VerifyNow assay was used to evaluate platelet function among ACS patients. Our results show that the mutant allele C was more frequent among ACS ST (+) than ST (−) patients (39% versus 19.8%, resp.), when the wild-type allele was more represented in the ACS ST (−) group (80.2%). The C allele frequency was higher among resistant than nonresistant patients (30% versus 20.8%, resp.). Comparison of ACS patients and healthy controls shows higher frequency of mutant C allele among cases compared to controls (22.73% versus 19.31%, resp.); there was a statistically significant association of the recessive and additive transmission models with the ACS development risk (OR [95% CI] = 1.78 [1.58–5.05], *P* = 0.01 and OR [95% CI] = 1.23 [0.74–2.03], *P* < 0.001, resp.), increasing thus the association of this polymorphism with the pathology.* Conclusion.* Our results suggest that this polymorphism may have a potential effect on Clopidogrel response among our Moroccan ACS patients and also on ACS development.

## 1. Background

Because the majority of cardiovascular diseases are the result of an occlusive thrombosis, numerous antithrombotic drugs are used in there therapy, including platelet inhibitors and oral anticoagulants [1].

Clopidogrel is a thienopyridine derivative, used to inhibit the formation of blood clots in coronary artery disease, peripheral vascular disease, and stroke. It irreversibly inhibits the receptor of adenosine diphosphate (ADP), called P2Y12, expressed on the surface of platelets. This prodrug requires a hepatic oxidation step by the cytochrome P450 (CYP450) enzymes, to generate the active metabolite responsible for the irreversible blocking effect of the P2Y12 receptor during the life of the platelet [2].

In Acute Coronary Syndrome (ACS), a wide range of interindividual variations in platelet response to Clopidogrel, has been described. An important proportion of patients still experience thrombotic events even after receiving the treatment, so they do not reach the same degree of benefit from the given drug [3]. Several factors were found to be in association with this heterogeneity in response to antithrombotic agents among patients [4, 5]. The role of pharmacogenomics is to study the genetic factors that determine the response of a given individual to a given drug. This variability of response to treatment may explain both its efficacy and its adverse side effects [6].

Several genes are involved in the modulation of this response. These genes may act in absorption of the molecule: transportation (ABCB), metabolism (cytochromes), excretion (for side effects), or targets of direct or indirect action of the molecule (receptors: P2Y12, Gp IIb/IIIa, Gp Ia/IIa, etc.) [7–15].

*P2Y12.* The P2Y12 is the platelet receptor for adenosine diphosphate (ADP) targeted by the active form of Clopidogrel [16]. The protein encoded by this gene belongs to the big family of G-protein coupled receptors, which contains several receptor groups with different pharmacological selectivity. It is involved in platelet aggregation and is a potential target for the treatment of thromboembolic pathologies and clotting disorders. The P2Y12 gene is localized on human chromosome 3 (3q25.1); it covers 47.97 pb of length. Mutations in this gene are implicated in bleeding disorder, platelet type 8 (BDPLT8). Many studies have assessed the functional role of the P2Y12 gene variants in modulating the response to antiplatelet drugs [11, 17]. Recently, the T744C polymorphism of the P2Y12 receptor gene has been associated with enhanced platelet aggregation in healthy volunteers, suggesting a possible mechanism for modulation of Clopidogrel response [18, 19].

The main objectives of our study is to determine the frequency of i-T744C P2Y12 polymorphism among Moroccan ACS and healthy subjects and to assess whether or not the Clopidogrel response may be influenced by this genetic polymorphism in a sample of Moroccan ASC patients.

## 2. Materials and Methods

### 2.1. Study Population

Patients were eligible for inclusion if they had documented antiplatelet therapy (Clopidogrel), a VerifyNow P2Y12 platelet function test, and no more heparin in their blood. Patients were excluded if they did not have a VerifyNow P2Y12 platelet function test or having incomplete clinical data. All patients received a baseline P2Y12 platelet function test to identify Clopidogrel resistance and determine whether they would need another loading dose to achieve P2Y12 response (PRU PRU < 208 and inhibition% ≥ 20%).

Blood samples were collected from 77 unrelated ACS Moroccan patients and 101 apparently healthy subjects showing no symptoms of coronary artery diseases. Clinical data concerning risk factors, biological parameters, and the VerifyNow test results were collected; an informed consent that was approved by the Ethical Committee of the University of Hassan II, School of Medecine, Casablanca, was signed by each patient and control before entering the study.

### 2.2. Study Protocol

Recruited patients received 300 mg loading dose of the generic molecule; it was replaced by 75 mg maintenance dose of Plavix for 7 days (washout period) if Clopidogrel resistance was noted on the initial platelet test (PRU > 208); a 300 mg loading dose of Plavix was prescribed if the PRU remains and the resistance persists; inhibition was considered adequate for good response to treatment if the value reached ≥20%.

The VerifyNow test (Accumetrics Inc., San Diego, California) was used to evaluate platelet function, as it is a point-of-care assay, easy to perform, and rapid and uses small volumes of whole blood samples [20]. Two results are reported: the PRU (P2Y12 Reactive Units) and the percent inhibition. The ideal percent of platelet inhibition is ≥30% for Clopidogrel; however, 20–30% inhibition is considered as intermediate response [21, 22]. In our study, resistance to Clopidogrel was defined by <20% inhibition + PRU > 208 after many platelet function tests and nonresistance by ≥20% inhibition + PRU < 208.

### 2.3. DNA Extraction

Venous blood from all participants in this study was collected in EDTA tubes. Samples were treated immediately or stored at −20°C until extraction of DNA. Genomic DNA was extracted from blood leukocytes using the standard method of salting out [23].

### 2.4. Genotype Determination

We used PCR-RFLP to genotype samples for i-T744C P2Y12 polymorphism, as previously described by Malek et al. [16]. Genotyping of this variant was performed by amplification from 50 to 100 ng of genomic DNA, followed by digestion using* RsaI* restriction enzyme. The digestion gave rise to three profiles: wild TT homozygous (one fragment of 220 pb), TC heterozygote (two fragments of 220 and 196 bp), and mutated CC homozygous (one fragment of 196 bp). The digested product was separated on 3% agarose gel electrophoresis stained with Ethidium Bromide (BET) and visualized with UV rays.

### 2.5. Statistical Analysis

Statistical analysis was performed using SPSS 21.0 software. Chi square test (*χ*^2^) was used to determine statistical significance of association/nonassociation between genotypes and classical risk factors. Hardy–Weinberg equilibrium (HWE) test was performed for cases and controls groups. Odds ratio (OR) were calculated to estimate the association between genotypes and ACS risk, using a Confidence Interval (CI) of 95%. Significance was approved at *P* value less than 0.05.

## 3. Results

### 3.1. Characteristics of the Study Population

The distribution of i-T744C P2Y12 polymorphism was in Hardy–Weinberg equilibrium (HWE) for controls and cases groups (Table 1). The average age was 57.33 ± 9.7 for patients versus 32 ± 9.87 for healthy controls. There was a predominance of male in both groups (cases: 54.54%; controls: 53.52%) (Table 2).  Table 3 describes the routine pathology data for our 77 SCA patients; 79.6% of these patients were under IPP, when 20.4% were not.

### 3.2. VerifyNow Results versus Risk Factors

Patients were placed into resistant and nonresistant groups, based on their platelet function test results, and the baseline characteristics of these patients correlated to resistance groups are shown in Table 4: only creatinine level, fibrinogen, Pq numerisation, and IPP use show statistically significant association (*P* = 0.01; 0.04; 0.04; and 0.03, resp.).

### 3.3. Allelic Frequencies

When correlating i-T744C P2Y12 genotypes to the classical risk factors of the pathology, a statistically significant association was found with familial antecedent among SCA patients; no association was detected with the other risk factors (Table 5).

Table 6 shows the distribution of patients ACS type (ST (+) and ST (−)) according to i-T744C P2Y12 genotypic profiles: 62.75% of the wild-type and 76.48% of the heterozygous profiles were SCA ST (−), when the majority of the mutated profile was SCA ST (+) (66.7%). Mutant allele was more frequent among SCA ST (+) than SCA ST (−) patients (39% versus 19.8%, resp.); the wild-type allele was more frequent in SCA ST (−) group than SCA ST (+) one (80.2% versus 61%, resp.).

Distribution of resistant and nonresistant patients according to i-T744C P2Y12 genotypes is reported in Table 7: 69.45% of nonresistant patients are wild-type for this polymorphism; 19.45% are heterozygous; and 11.1% are homozygous for the mutation; in the resistant group, 60% are wild-type, 20% are heterozygous, and 20% are homozygous mutant. The mutant allele is more frequent among resistant than nonresistant patients (30% and 20.8%, resp.).

Allelic and genotypic frequencies among cases and controls are reported in Table 8: genotypic frequencies were 66.23% TT, 20.07% TC, and 11.7% CC among cases versus 68.32% TT, 24.75% TC, and 6.93% CC among healthy controls. Allelic frequencies were 77.27% T and 22.73% C among cases versus 80.69% T and 19.31% C among controls. A statistically significant association was found with both TC and CC genotypes (OR [95% CI] = 0.92 [0.45–1.87], *P* = 0.0048 and OR [95% CI] = 1.74 [1.66–5.00], *P* = 0.03, resp.). There was a positive correlation with the recessive and additive transmission models, but not the dominant one (OR [95% CI] = 1.78 [1.58–5.05], *P* = 0.01 and OR [95% CI] = 1.23 [0.74–2.03], *P* < 0.001, resp.), increasing thus the association of this polymorphism with the pathology.

## 4. Discussion

Clopidogrel is a second-generation thienopyridine, having better efficacy of ticlopidine that represents the first-generation; it has better tolerability profiles and is currently the antiplatelet treatment of choice for prevention of thrombosis events [24, 25]. An interindividual variation in platelet response to Clopidogrel has been widely reported; it may be explained by several factors, including genetics [6]. Many Single Nucleotide Polymorphisms (SNPs) of the P2Y12 receptor gene were described to be in association with this interindividual variability in ADP-induced platelet aggregation [26–28]. i-T744C polymorphism has been associated with enhanced platelet aggregation, suggesting its potential effect on modulating Clopidogrel response [29, 30].

Our study is the first to determine the frequency of i-T744C P2Y12 polymorphism among Moroccan ACS patients and healthy subjects and to evaluate the correlation between Clopidogrel resistance and genetic testing represented by i-T744C P2Y12 polymorphism, among a sample of Moroccan ACS patients.

In our study sample, there was a predominance of male in both cases and controls groups (54.54% and 53.52%, resp.); the average age was 57.33 and 32, respectively, among cases and controls (Table 2). This was in agreement with what Zoheir et al. found in their study about P2Y12 receptor gene polymorphism and antiplatelet effect of Clopidogrel in patients with coronary artery disease after coronary stenting [26].

79.6% of our patients were under PPI, when 20.4% were not (Table 3). PPI use showed a statistically significant association when being correlated to patients groups (resistant and nonresistant patients), divided based on their platelet function test results (*P* = 0.03) (Table 4). Proton-pump inhibitors (PPI) are known to potentially affect the Clopidogrel platelet inhibition relationship [30]; also creatinine level, fibrinogen, and Pq numerisation showed statistically significant association (*P* = 0.01; 0.04; and 0.04, resp.) (Table 4).

Concerning resistant and nonresistant groups of patients, our results were in agreement with Nordeen et al.'s study: they found the same range of age, with the majority of resistant group being female, compared to nonresistant one [30]. Several studies have suggested that women do not accrue equal therapeutic benefit of antithrombotic therapy [31, 32]. Although multiple contributing factors have been described (differences in vessel wall biology between men and women; the direct influence of sex hormones (oestrogens, progesterone, or androgens) on platelets and their indirect effect on the vasculature), the physiological mechanism behind this gender disparity remains unclear [33].

A statistical comparison was held between distribution of i-T744C polymorphism among ACS patients and traditional risk factors; we found significant association only with familial antecedent factor (*P* = 0.014; Table 5). Correlation between this polymorphism and ACS subgroups (ST+ and ST−) showed that the majority of wild-type and heterozygous profiles were SCA ST (−), when the majority of the mutated profiles were SCA ST (+) (Table 6). Mutant allele was more frequent among SCA ST (+) patients, when wild-type allele was more present in SCA ST (−) group. Distribution of resistant and nonresistant patients according to i-T744C P2Y12 genotypes showed that 69.45% of nonresistant patients had the wild-type profile; 19.45% were heterozygous; and 11.1% were homozygous mutant; in the resistant group, 60% were wild-type, 20% heterozygous, and 20% homozygous mutant. The mutant allele was more frequent among resistant than nonresistant patients (30% and 20.8%, resp.). Zoheir et al. [26] found a higher expression of C allele (heterozygous CT and homozygous CC) among nonresponder ACS patients (*P* < 0.001). Fontana et al. [29] also found similar results suggesting that the H2 haplotype of the P2Y12 gene is associated with increased platelet function in nonmedicated healthy volunteers. On the contrary, the results of Cuisset et al. [18] show no influence of i-T744C P2Y12 polymorphism on Clopidogrel response. Platelet function studies performed by Lev et al. [12] in the same context did not show any modulating effect of this genetic polymorphism on individual responsiveness to Clopidogrel. Furthermore, Hetherington et al. [34] reported no significant effect of i-T744C P2Y12 SNP on platelet response to ADP among subjects without cardiovascular disease history.

In our study, we tried also to investigate whether the mutant allele C of i-T744C P2Y12 polymorphism has an effect on ACS occurrence. Our results revealed that the mutant allele C was more frequent among cases than controls (22.73% versus 19.31%, resp.). A statistically significant association was found with both TC and CC genotypes (OR [95% CI] = 0.92 [0.45–1.87], *P* = 0.0048 and OR [95% CI] = 1.74 [1.66–5.00], *P* = 0.03, resp.). There was a positive correlation between the recessive and additive transmission models and ACS risk, but not the dominant one (OR [95% CI] = 1.78 [1.58–5.05], *P* = 0.01 and OR [95% CI] = 1.23 [0.74–2.03], *P* < 0.001, resp.), increasing thus the association of this polymorphism with the risk of pathology development. Our findings matches those of Zoheir et al. [26]; they reported that this polymorphism was positively correlated to increased risk of disease development (OR = 14.8, 95%, CI = 1.8–121.1, and *P* = 0.002). Similar findings were published by Cavallari et al. [35], who found that nonsmokers carrying the minor haplotype H2 of the gene were highly associated with significant CAD (OR = 1.83, 95% CI = 1.17–2.87, and *P* = 0.007). On the other side, findings of Schettert et al. [36] did not provide evidence for a strong association between H1/H1 and H1/H2 haplotypes and any increased risk of cardiovascular events in a population with CAD.

## 5. Conclusion

To the best of our knowledge, our study is the first in Morocco to assess whether or not Clopidogrel response may be modulated by i-T744C P2Y12 polymorphism in a sample of Moroccan ACS patients. We tried also to determine the frequency of this polymorphism among Moroccan ACS and healthy subjects. Our findings suggest that the wild-type and heterozygous genotypic profiles were more frequent among ACS ST (−) patients, when the homozygous mutant genotype was more frequent among ACS ST (+). The mutant allele C was more frequent among ACS ST (+) than ACS ST (−) patients, when the wild-type allele was more represented in the ACS ST (−) group. The C allele frequency was higher among resistant than nonresistant patients. Comparison of ACS patients and healthy controls shows higher frequency of C allele among cases than controls; there was a statistically significant association of the recessive and additive transmission models with the ACS development risk, increasing thus the association of this polymorphism with the pathology. Further studies including larger sample sizes and exploring interactions between this polymorphism and others are still be needed and may provide useful information to better understand the mechanism of Clopidogrel interindividual resistance and also the risk of ACS occurrence in the option to improve the biomedical context.

## Figures and Tables

**Table 1:** HWE among cases and controls.

Genotypes	EHW cases		EHW controls	
	*χ* ^2^ square	*P* value (*P* > 0.05)	*χ* ^2^ square	*P* value (*P* > 0.05)
i-T744C P2Y12 (rs2046934)	1.35	0.51^*∗*^	4.26	0.13^*∗*^

^*∗*^Statistically significant.

**Table 2:** Description of ACS study population.

	ACS patients	Controls
*Age (years)*	57.33 ± 9.7	32 ± 9.87
*Age of disease occurrence (years)*	54.81 ± 10.2	
*Sex*		
Male	54.54%	53.52%
Female	45.46%	46.48%
*Ethnicity*		
Arab	88%	86%
Berber	2%	4%

**Table 3:** Routine pathology data of our ACS patients.

Parameters	SCA patients
Total cholesterol (g/L)	1.88 ± 0.73
HDL (g/L)	1.24 ± 0.9
LDL (g/L)	1.24 ± 0.64
Triglycerides TG (g/L)	1.53 ± 1.00
Glucose (g/L)	1.43 ± 0.8
Creatinine (mg/L)	9.51 ± 2.46
Fibrinogen	3.65 ± 1.06
HB (g/dL)	13.99 ± 2.63
GB (elts/mm^3^)	12958.6 ± 24041.3
Pq (elts/mm^3^)	237151.7 ± 102412
BMI (Kg/m^2^)	26.72 ± 4.17
IPP	
(+)	79.6%
(−)	20.4%

**Table 4:** Baseline characteristics of our SCA patients versus VerifyNow test results.

	Nonresistant%	Resistant%	*P* value
*Age *	56.9 ± 8.48	62 ± 8.02	0.9
*Sex*			0.41
Male	58.3%	20%	
Female	41.7%	80%	
*SCA type*			0.2
*ST (*+)	36%	60%	
*ST (*−)	64%	40%	
*Familial ACD *			0.8
Presence	2.8%	0%	
Absence	97.2%	100%	
*Personal ACD*			0.3
Presence	41.7%	20%	
Absence	58.3%	80%	
*Diabetes*			0.6
Presence	40.3%	40%	
Absence	59.7%	60%	
*HTA*			0.46
Presence	47.2%	60%	
Absence	52.8%	40%	
*Dyslipidemia*			0.22
Presence	16.7%	40%	
Absence	83.3%	60%	
*Smoking*			0.6
Presence	40.3%	40%	
Absence	59.7%	60%	
*Creatinine (mg/L)*	11.6 ± 10.87	15 ± 4.34	0.01^*∗*^
*Fibrinogen*	3.57 ± 1.06	5.03 ± 1.14	0.04^*∗*^
*Pq*	242333 ± 102412	159427 ± 79609	0.04^*∗*^
*IPP*			0.03^*∗*^
Used	50.38%	60%	
Nonused	49.62%	40%	

^*∗*^Statistically significant.

**Table 5:** i-T744C P2Y12 rs2046934 genotypes distribution versus risk factors.

Risk factor	SCA patients			*P* value (<0.05)
	Wild-type%	Heterozygous%	Mutant%	
*Familial ACD *				0.014^*∗*^
Presence	0	100	0	
Absence	71.4	17.2	11.4	
*Personal ACD *				0.6
Presence	75	17.9	7.1	
Absence	65.9	20.5	13.6	
*HTA*				0.8
Presence	71.4	17.2	11.4	
Absence	67.6	21.6	10.8	
*Smoking*				0.51
Presence	70	23.3	6.7	
Absence	69	16.7	14.3	
*Diabetes*				0.71
Presence	65.5	24.2	10.3	
Absence	72.1	16.3	11.6	
*Dyslipidemia*				0.8
Presence	71.4	21.4	7.2	
Absence	69	19	12	

^*∗*^Statistically significant.

**Table 6:** i-T744C P2Y12 rs2046934 genotypes distribution VS ACS subgroups.

	SCA patients			Wild-type allele	Mutant allele	*P* value (<0.05)
	TT%	TC%	CC%			
ST (+)	37.25	23.52	66.7	61%	39%	0.26
ST (−)	62.75	76.48	33.3	80.2	19.8%	

**Table 7:** i-T744C P2Y12 rs2046934 genotypes distribution versus VerifyNow test results.

	SCA patients			Wild-type allele%	Mutant allele%	*P* value
	TT%	TC%	CC%			
Nonresistant	69.45	19.45	11.1	79.2	20.8	0.45
Resistant	60	20	20	70	30	

**Table 8:** Allelic and genotypic frequencies of i-T744C P2Y12 rs2046934 polymorphism among cases and controls.

	Genotypes/alleles	Cases (%)	Controls (%)	OR (95% CI)	*P* value
i-T744C P2Y12 (rs2046934)	TT	66.23	68.32	1	
	TC	22.07	24.75	0.92 [0.45–1.87]	0.0048^*∗*^
	CC	11.7	6.93	1.74 [1.66–5.00]	0.03^*∗*^
	TT + TC^(b)^	88.3	93.07	1	
	CC	11.7	6.93	1.78 [1.58–5.05]	0.01^*∗*^
	TT^(c)^	66.23	68.32	1	
	TC + CC	33.77	31.68	1.1 [1.7–2.05]	0.15
	T^(d)^	77.27	80.69	1	
	C	22.73	19.31	1.23 [0.74–2.03]	<0.001^*∗*^

^*∗*^Statistically significant.

^b^Recessive model.

^c^Dominant model.

^d^Additive model.
